# Are birth outcomes in low risk birth cohorts related to hospital birth volumes? A systematic review

**DOI:** 10.1186/s12884-021-03988-y

**Published:** 2021-07-27

**Authors:** Felix Walther, Denise Kuester, Anja Bieber, Jürgen Malzahn, Mario Rüdiger, Jochen Schmitt

**Affiliations:** 1 Center for Evidence-Based Healthcare, Medical Faculty Carl Gustav Carus, TU Dresden, Fetscherstraße 74, 01307 Dresden, Germany; 2 Quality and Medical Risk Management, University Hospital Carl, Gustav Carus, Fetscherstraße 74, 01307 Dresden, Germany; 3 Institute of Health and Nursing Science, Medical Faculty, Martin Luther University Halle-Wittenberg, Postfach 302, 06097 Halle, Saale Germany; 4 Federation of Local Health Insurance Funds, Clinical Care, Rosenthaler Str. 31, 10178 Berlin, Germany; 5 Department for Neonatology and Pediatric Intensive Care, University Hospital Carl Gustav Carus, Technische Universität Dresden, Fetscherstraße 74, 01307 Dresden, Germany; 6 Medical Faculty Carl Gustav Carus, Saxony Center for Feto-Neonatal Health, TU Dresden, Fetscherstraße 74, 01307 Dresden, Germany

**Keywords:** Mortality, Infant, Low risk birth, Perinatal regionalization, Volume-outcome

## Abstract

**Background:**

There is convincing evidence that birth in hospitals with high birth volumes increases the chance of healthy survival in high-risk infants. However, it is unclear whether this is true also for low risk infants. The aim of this systematic review was to analyze effects of hospital’s birth volume on mortality, mode of delivery, readmissions, complications and subsequent developmental delays in all births or predefined low risk birth cohorts. The search strategy included EMBASE and Medline supplemented by citing and cited literature of included studies and expert panel highlighting additional literature, published between January/2000 and February/2020. We included studies which were published in English or German language reporting effects of birth volumes on mortality in term or all births in countries with neonatal mortality < 5/1000. We undertook a double-independent title-abstract- and full-text screening and extraction of study characteristics, critical appraisal and outcomes in a qualitative evidence synthesis.

**Results:**

13 retrospective studies with mostly acceptable quality were included. Heterogeneous volume-thresholds, risk adjustments, outcomes and populations hindered a meta-analysis. Qualitatively, four of six studies reported significantly higher perinatal mortality in lower birth volume hospitals. Volume-outcome effects on neonatal mortality (*n* = 7), stillbirths (*n* = 3), maternal mortality (*n* = 1), caesarean sections (*n* = 2), maternal (*n* = 1) and neonatal complications (*n* = 1) were inconclusive.

**Conclusion:**

Analyzed studies indicate higher rates of perinatal mortality for low risk birth in hospitals with low birth volumes. Due to heterogeneity of studies, data synthesis was complicated and a meta-analysis was not possible. Therefore international core outcome sets should be defined and implemented in perinatal registries.

**Systematic review registration:**

PROSPERO: CRD42018095289

**Supplementary Information:**

The online version contains supplementary material available at 10.1186/s12884-021-03988-y.

## Background

Several studies have shown mortality of high-risk-infants can be reduced if these infants are treated in highly equipped neonatal intensive or intermediate care units [1]. Therefore, different levels of care have been introduced for treatment of pregnant women and their newborns in relation to the medical condition. For each level certain requirements in terms of infrastructure, staffing, equipment and qualifications are defined. If a centre does not fulfill these requirements, a specialized care is usually not allowed [2, 3]. Since experience of the care team is likely to be also of advantage, it could be assumed that infants will benefit from hospitals with high annual birth volume. That assumption is supported by our recent systematic review, showing for very low birth weight infants an improved maternal and neonatal outcome in centers with higher birth volumes in high-risk births [4].

Important other risk factors for pregnancy and birth complications are higher maternal age, comorbidities (e.g. placenta praevia, pre-existing or gestational diabetes) or smoking. These factors are likely to increase the risks for maternal or neonatal adverse events [5–10]. Currently, appropriate management of these risks is still being discussed [11–15]. In order to better study the impact of different interventionson on subsequent outcome, a homogenous definition of birth outcomes is needed and core outcome sets (COS) are currently developed [5, 6]. COS are multilaterally consented and standardized sets of outcomes which should be reported in clinical trials to guarantee comparabilityIn recent years, COS have been increasingly developed and registered for perinatal and maternal care [16], like gestational diabetes [17], preterm birth [18], maternity care [19], neonatal medicine [20] or pregnancy and childbirth [21]. However, currently there are no COS available to study the impact of birth volume on outcome of low risk pregnancies. For both this reason and since birth complications are difficult to predict in low risk pregnancies, it remains unknown whether women with a low risk pregnancy could also benefit from care in hospitals with higher birth volumes.

The aim of this systematic review was to summarize and critically appraise the impact of hospital case volume on mortality and morbidity in low risk birth cohorts.

## Methods

We conducted this systematic review in accordance with the Preferred Reporting Items for Systematic Reviews and Meta-Analyses (PRISMA) Checklist [22] and registered the review protocol (CRD42018095289) in the International Prospective Register of Systematic Reviews [23]. The original search strategy (Additional file 1) and review was designed to identify studies on the effects of either perinatal regionalization or hospital birth volume on infant and maternal outcomes. Here we report on the results of volume-outcome-relationships.

### Eligibility criteria, information sources, search strategy

Inclusion and exclusion criteria (Table 1) addressed population, intervention, comparison, outcome and study type (PICOS). *Interventions/ expositions* included volume effect estimates on mortality as primary outcome and secondarily on caesarean sections, readmissions, birth complications, developmental delays *(outcome)* in all births or a pre-defined low risk birth cohort *(population)*. In order to ensure comparability and current status of obstetric care, observational or interventional studies *(study type)* from countries with neonatal mortality rates below 5 per 1000 births (UN Child mortality report) that were published in English or German language after 01/01/2000 were included [24].

**Table 1:** PICO-Scheme

	**Inclusion criteria**	**Exclusion criteria**
POPULATION	all births, term/ normal birth weight birth or low risk birth in a nationwide setting with < 5/1000 neonatal deaths	Preterm birth, low birth weight birth, other risk-selections (e.g. gestational diabetes, multiple births)
EXPOSITION	comparison of different hospital birth volumes or -sizes	No comparison of different hospital birth volumes or -sizes
COMPARISON	other birth volumes	No comparator provided
OUTCOME	Primary Outcome: Maternal or infant mortality Secondary Outcomes: Caesarean sections, readmissions, birth complications, developmental delays	No measurement of maternal or infant mortality
STUDY TYPE	Observational and interventional studies	Descriptive studies, systematic reviews

### Study selection

We systematically searched Medline and EMBASE on 18/04/2018 and on 26/02/2020. The search strategy included a combination of free text words and database-specific subject-headings (Additional file 1) using Ovid interface. We used Endnote X7 for the creation of the literature database and the removal of duplicates. Two authors (FW, AB) independently screened titles/ abstracts and full texts for eligibility. Additionally, an expert panel (MR, JM, Rainer Rossi) highlighted missing relevant papers. After full-text-screening, we conducted a hand search including forward (citing literature) and backward (cited literature) screening of included studies. Discrepancies during screening, extraction or quality assessment were solved by consulting of another reviewer (JS). For interpretation of reliability, we applied the prevalence-adjusted bias-adjusted kappa (PABAK). The advantage of PABAK in contrast to Kappa value is the consideration of the high class imbalance [25].

### Data extraction and data synthesis

We predefined a data extraction form in MS Excel including study charateristics (e.g. population, period, country) and outcomes (e.g. definition, exposing/ referencing annual volume, result, estimator) was used. One reviewer extracted (FW) and a second (DK) verified the results resolving discrepancies by consensus or consulting a third reviewer (JS). To decide whether individual studies can be pooled in a meta-analysis, we reviewed methodological quality, comparability of the study contexts (population, outcomes, volume-thresholds and risk adjustment) and statistical heterogeneity. If studies were considered as not comparable, a qualitative synthesis followed.

### Critical appraisal process

Two independent reviewers (FW, DK) performed the quality assessment using the Methodology Checklist for Cohort studies of the Scottish Intercollegiate Guidelines Network (SIGN). This checklist contains 14 items with a final quality rating of the studies in "high quality", "acceptable" and "inacceptable" [26]. Methodological explanations and definitions in the context of the application of the checklist are presented in Additional file 2.

### Patient and public involvement

No patient involved.

## Results

### Study selection

**Fig. 1:**
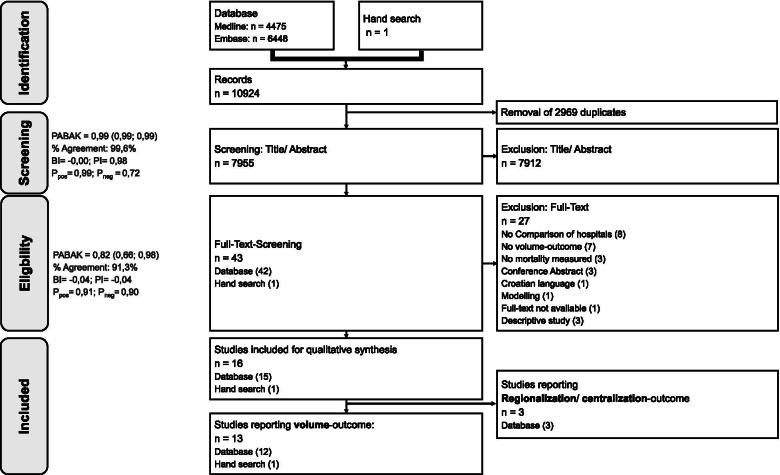
PRISMA flow-chart

After screening of 7955 records 13 studies met our predefinded eligibility criteria were included in the systematic review (Fig. 1) [27–39]. Additional file 3 contains the reasons for exclusion of the remaining 30 full texts [40–69]. The high prevalence and bias adjusted Kappa (PABAK) (Fig. 1) in both title-abstract and full-text-screnning suggests no systematic differences between the raters.

### Study characteristics

Table 2 shows the characteristics of included studies. The observation period varied between 29 years (1967–1996) [33] and one year [35, 39]. The earliest observation started in 1967 [33] and the latest ended 2012 [39]. All of the included studies used cross-sectional designs to analyse retrospective cohorts in perinatal registers (Additional file 4). The studies were conducted in Finland [30, 32, 34], the United States [28, 35, 39], Sweden [27], Norway [33], Germany, [29] the United Kingdom, [31] Australia, [36] the Netherlands [70] and Canada [37]. The analyzed populations consist of either all births [27, 28, 30, 31, 33–35, 37, 39] and/ or a predefined low risk population [29, 32, 34, 36, 38] excluding e.g. low birth weight or multiple births. Annual volumes and its comparators were set differently in terms of group sizes and defining births [27, 29–33, 36, 39] or deliveries/ pregnancies respectively women giving birth [28, 34, 35, 37, 38] as basis for the calculation. While “birth” refer to the neonate, “delivery” describes the mother who is giving birth. Due to multiple pregnancies, number of deliveries is usually lower than the number of births. Unfortunately, not all studies reported both numbers, but Table 2 shows the different annual volumes in the included studies. In addition to the different annual volumes, maximum, [29, 33, 36–39] minimum [35] and mean quantities [27, 28, 34] as well as university clinics (UH) [30, 32] were used as reference volumes. The analyzed outcomes included stillbirths, [31, 32, 34] perinatal/ early [29, 30, 32, 34, 37, 38] and neonatal mortality, [27, 31, 33–36, 39] birth by caesarean section [30, 36] and composite outcomes like perinatal adverse outcome [38] or maternal morbidity/ mortality [37]. Six out of thirtheen studies did not solely focus on volume-outcome relationship, but analyzed influence of geographic accessibility [37], birth at night hours [38], staffing [31], availability of facilities [31], on call arrangements [32], or birth at weekday/ weekend [39].

**Table 2:** Characteristics of included studies

Study	Period	Country	Birth population	Grouped annual hospital volume	Outcomes	Outcome definition
Finnstrom et al. 2006[27]	1985–1999	SWE	births: all singletons (n = 1.538.814)	< 500, 500–999, 1000–2499 (ref.), ≥ 2500	1) neonatal mortality	1) ≤ 27d
Friedman et al. 2016[28]	1998–2010	US	women: all hospital (n = 50.433.539)	50, 1000 (ref.), 1500, 2250	1) maternal mortality 2) maternal complications	1) failure to rescue 2) severe morbidity^1^
Heller et al. 2002[29]	1990–1999	GER	births: BW > 2500 g (n = 582.655);	≤ 500, 501–1000, 1001–1500, > 1500 (ref.)	1) Early-neonatal death	1) ≤ 7d
Hemminki et al. 2011[30]	1991–2008	FIN	births: all (n = 474.419) + BW > 2499 g in non-UH	< 750, 750–1499, ≥ 1500, UH (ref.)	1) perinatal mortality 2) CS	1) ≤ 7d
Joyce et al. 2004[31]	1994–1996	UK	births: all (n = 540.834)	N/A: Volume entered the analysis as continuous variable	1) stand. stillbirth rates 2) stand. neonatal mortality	1) > 24 wk GA 2) ≤ 28d
Karalis et al. 2016[32]	2005–2009	FIN	births: low risk^2^ (n = 276.066)	births: ≤ 999, 1000–1999, ≥ 2000, UH (ref.)	1) stillbirths 2) early neonatal death	1) Intrapartum: undefined 2) undefined
Moster et al. 2001[33]	1967–1996	NO	births: all (n = 1.650.852)	≤ 100, 101–500, 501–1000, 1001–2000, 2001–3000, > 3000 (ref.)	1) neonatal mortality	1) ≤ 28d
Pyykonen et al. 2014[34]	2006–2010	FIN	women: all^3^ (n = 290.288) + low risk^4^ (n = 276.287)	< 1000, 1000–2999 (ref.), < 3000	1) perinatal mortality 2) neonatal mortality 3) early neonatal mortality 4) stillbirths	1) stillbirth + death ≤ 7d 2) ≤ 28d 3) ≤ 7d 4) ≥ 22wk GA
Snowden et al. 2012[35]	2006	US	women: all (n = 527.617), low risk^5^	Urban: ≤ 50–1199 (ref.), 1200–2399, 2400–3599; ≥ 3600 Rural: 50–599 (ref.) 600–1699; ≥ 1700	1) neonatal mortality	1) undefined
Tracy et al. 2006[36]	1999–2001	AUS	births: low risk/ term^6^ (n = 331.147)	< 100, 100–500, 501–1000, 1001–2000, > 2001 (ref.)	1) neonatal mortality 2) CS (labour) 3) Overall CS	1) ≤ 28d
de Graaf et al. 2010[38]	2000–2006	NEL	women: singleton (n = 655.961)	< 750, 750–999, 1000–1249, 1250–1499, 1500–1749, ≥ 1750 (ref.)	1) perinatal mortality 2) neonatal complications	1) ≤ 7d 2) Perinatal adverse outcome^7^
Restrepo et al. 2018[39]	2012	US	births: live 20–44 wk GA (n = 32.140)	N/A: Volume entered the analysis as continu-ous variable	1) neonatal mortality	1) ≤ 28d
Aubrey-Brassler et al. 2019[37]	2006–2009	CA	women: all (n = 820.761)/ births: all (n = 827.504)	No services usually; 1–49; 50–99; 100–199; 200–499; 500–999; 1000–2499, > 2500 (ref.)	1) perinatal mortality 2) maternal complications	1) Death […]^8^ 2) Maternal Morbidity & Mortality^9^

Notes:

1: heart/ renal/ respiratory failure, acute myocardial infarction, liver disease, disseminated intravascular coagulation, coma, delirium, puerperal cerebrovascular disorders, pulmonary edema or embolism, sepsis, shock, status asthmaticus, status epilepticus

2; Exclusion: Low BW, multiple pregnancy, antepartum stillbirth, out-of-hospital birth, major congenital anomalies, birth defects

3: Exclusion: birth in university hospital, length of stay > 7d

4: Exclusion: birth in university hospital, length of stay > 7d, multiple pregnancy, pre-/postterm birth

5: Exclusion: preterm birth, low BW

6: Exclusion: Low BW, multiple pregnancy, preterm, age, complications

7: intrapartum death, death ≤ 7d, 5-min Apgar < 7, NICU transfer

8: sudden infant death syndrome, sudden cardiac death, stillbirth (GA ≤ 20 wk), in-hospital death liveborn neonate

9: Eclampsia, Previa with hemorrhage abruption, Intrapartum + postpartum hemorrhage + transfusion or hysterectomy, Rupture of uterus before or during labor, Obstetric shock, Sepsis, Other complications of obstetric procedures, Obstetric embolism, Cardiovascular disease, Acute renal failure, Death, obstetric or unspecified, Neurologic disease, Hematologic disease, Respiratory disease, Diabetic ketoacidosis, Peritonitis or parametritis, Toxic liver disease or hepatic failure, Canadian Classification of Health Interventions, Assisted ventilation or resuscitation, Dialysis, Hysterectomy, Evacuation of incisional hemato-ma, Repair of bladder, urethra or intestine, Embolization or ligation of pelvic vessels or suturing of uterus, Blood transfusion

## Results of the critical appraisal

Table 3 shows in detail that most of the included studies (12 out of 13 studies) fulfilled the majority of the queried items leading to an “acceptable” quality [27–32, 34–39]. Quality of one study was rated as “unacceptable” due to lack of comparability (missing baseline-tables, item 1.2) of the investigated groups [33].

**Table 3:** Detailed results of sign—quality assessment for cohort studies

**Item**	**Description**	Finnstrom et al. 2006 [27]	Friedman et al. 2016 [28]	Heller et al. 2002 [29]	Hemminki et al. 2011 [30]	Joyce et al. 2004 [31]	Karalis et al. 2017 [32]	Moster et al. 2001 [33]	Pyykonen et al. 2014 [34]	Snowden et al. 2012 [35]	Tracy et al. 2006 [36]	de Graaf et al. 2010 [38]	Restrepo et al. 2018 [39]	Aubrey-brassler et al. 2019[37]
1.1	appropriate and clearly focused question	Yes	Yes	Yes	Yes	Yes	Yes	Yes	Yes	Yes	Yes	Yes	Yes	Yes
1.2	illustrated comparability between studied groups	Yes	Yes	Yes	Yes	Yes	Yes	No	Yes	Yes	Yes	Yes	Yes	Yes
1.3	number of asked people *(prospective studies)*	N/A	N/A	N/A	N/A	N/A	N/A	N/A	N/A	N/A	N/A	N/A	N/A	N/A
1.4	Likelihood that some eligible subjects might have the outcome at the time of enrolment is assessed and taken into account in the analysis	N/A	N/A	N/A	N/A	N/A	N/A	N/A	N/A	N/A	N/A	N/A	N/A	N/A
1.5	Drop-Out rate *(prospective studies)*	N/A	N/A	N/A	N/A	N/A	N/A	N/A	N/A	N/A	N/A	N/A	N/A	N/A
1.6	Comparison between full and lost-to-follow-up participants *(prospective studies)*	N/A	N/A	N/A	N/A	N/A	N/A	N/A	N/A	N/A	N/A	N/A	N/A	N/A
1.7	Clearly defined outcomes	Yes	Yes	Yes	No	Yes	No	Yes	Yes	Yes	Yes	Yes	Yes	Yes
1.8	Assessment of outcome blinded to exposure status	No	No	No	No	No	No	No	No	No	No	No	No	No
1.9	When blinding impossible, recognition that knowledge of exposure status could have influenced assessment	No	No	No	No	No	No	No	No	No	No	No	No	No
1.10	reliable measurement of exposure	Yes	Yes	Yes	Yes	Yes	Yes	Yes	Yes	Yes	Yes	Yes	Yes	Yes
1.11	from other sources is used to demonstrate that the method of outcome assessment is valid and reliable (clearly defined primary outcomes)	N/A	N/A	N/A	N/A	N/A	N/A	N/A	N/A	N/A	N/A	N/A	N/A	N/A
1.12	Exposure level or prognostic factor is assessed more than once *(prospective studies)*	N/A	N/A	N/A	N/A	N/A	N/A	N/A	N/A	N/A	N/A	N/A	N/A	N/A
1.13	confounders identifed and adequately taken into account for analysis	Yes	Yes	Yes	Yes	Yes	Yes	Yes	No	Yes	Yes	Yes	Yes	Yes
1.14	confidence intervals provided	Yes	Yes	Yes	Yes	Yes	Yes	Yes	Yes	No	Yes	Yes	No	Yes
**2.1**	Overall rating	Acceptable	Acceptable	Acceptable	Acceptable	Acceptable	Acceptable	Unacceptable	Acceptable	Acceptable	Acceptable	Acceptable	Acceptable	Acceptable

Due to the retrospective design and other methodological reasons, some items were not applicable:

- number of participants (item 1.3)
- outcome already present before start of study (item 1.4)
- drop-out (item 1.5)
- comparison between full and lost to follow-up (item 1.6) and
- multiple measured exposure levels (item 1.12).

None of the studies fulfilled the criteria for blinding (item 1.8) and critical recognition of limited possibilities of blinding (item 1.9) in cohort studies. An externally demonstrated validity (item 1.11) and reliability (item 1.10) of the assessed outcomes was not applicable due mortality, caesarean sections or other clinical outcomes are not subjective measures.

We originally planned to perform a meta-analysis but were unable to conduct it due to definitional heterogeneities in the included studies. Additional file 5 provides a tabular overview of heterogeneities identified between the outcomes analyzed. Five studies were excluded from a pooled estimate due to singular report of the outcome maternal mortality, [28] maternal morbidity/ mortality, [37] neonatal complications, [38] missing adjustments [34, 35] and the singular use of risk ratios as estimator, [31] 99% confidence intervals [36] or pearson correlation coefficients [39]. The remaining results for the outcomes stillbirth, [32, 34] perinatal/ early neonatal mortality, [29, 30, 32, 37, 38] neonatal mortality [27, 33, 39] and caesarean sections [30] were not comparable due to heterogeneously defined adjustment variables, populations (all births vs. predefined low risks), outcomes (e.g. undefined vs. defined) and volume-thresholds. Consequently, we summarized the results qualitatively.

### Effects of annual volume on neonatal outcomes

**Fig. 2:**
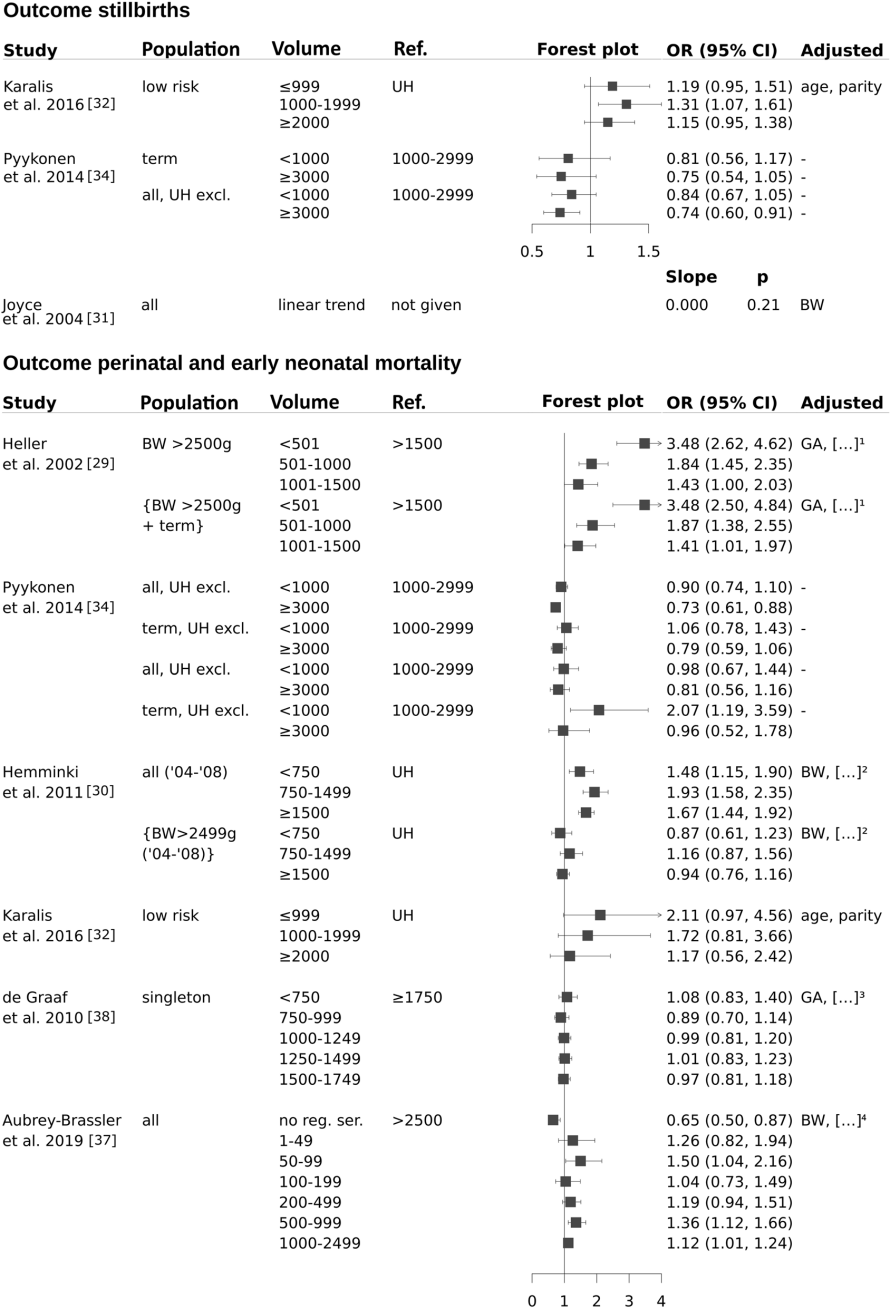
Stillbirths and early/ perinatal mortality. Legend: […]^1^ BW, age, parity, born outside clinic, birth planned and documented clinic, mode of delivery, born before arrival at clinic, time of birth, congenital anomaly/ malformation. […]^2^ age, parity, socio-economic position. […]^3^ age, parity, mode of delivery, ethnicity, calendar year trend. […]^4^ gender, Eclampsia, Premature rupture of membranes, Oligohydramnios, Abruptio placentae, Prolapsed umbilical cord, Noxious influences transmitted via placenta/ breast milk, Congenital anomalies, Hydrops fetalis, Other maternal conditions

Stillbirth was evaluated in three studies [31, 32, 34] and defined as fetal death prior to 22 [34] or 24 [31] weeks of gestation or remained undefined [32]. For hospitals with medium-sized birth volumes (1000–1999 p.a.) stillbirth odds ratio was significantly higher when compared with university hospitals [32]. Similar effects were found for hospitals with birth volumes between 1000–2999, when compared with high birth volumes (≥ 3000 p.a.) [34]. However, taking all data together there was no clear volume effect on the rate of stilbirths (Fig. 2).

Perinatal or early neonatal mortality has been defined as death within the first 7 days of life [29, 30, 34, 38] or as a combined outcome [34, 37]. One study did not provide a specific definitio [32]. Results were always adjusted, except for one study [34]. Whereas two studies did not report a significant volume-effect, [32, 38] four studies showed significantly higher rates of perinatal/ early neonatal mortality in hospitals with low (≤ 1000) [29, 30, 34, 37] or very low (≤ 500) [29, 37] birth volumes (Fig. 2) for either low risk (term infants with birthweight > 2499 g) [29, 34] or all births [30, 37].

**Fig. 3:**
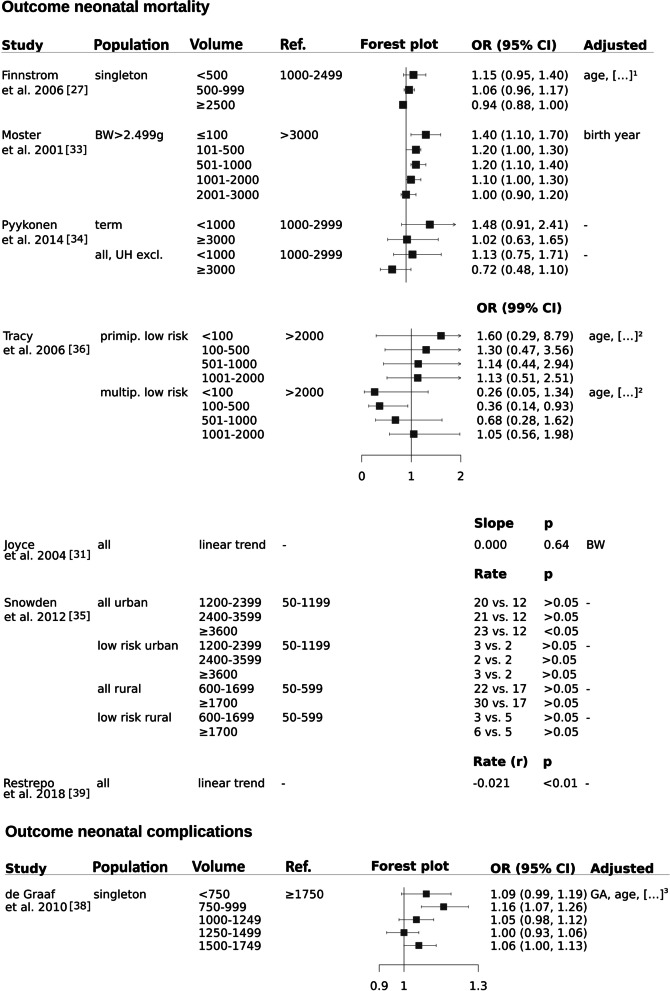
Neonatal complications and neonatal mortality. Legend: […]^1^ parity, GA, year of birth, smoking, parental cohabitation, maternal BMI. […]^2^ insurance status, maternal Aboriginal or Torres Strait Island status, maternal residential area. […]^3^ parity, mode of delivery, ethnicity, calendar year trend

Neonatal mortality was defined as 28-day-, [31, 33–36, 39] or 27-day-mortality [27] in order to analyze all [31, 33–36, 39] and/or low risk births [27, 34–36]. The majority of the studies undertook adjustments [27, 31, 33, 36]. As illustrated in Fig. 3 five [27, 33, 35, 36, 39] out of seven studies reported significant volume effect estimates with neonatal mortality being higher in hospitals with lower [33] or higher annual birth volumes [27, 35, 36, 39]. The remaining two studies reported non-significant volume-outcome effects [31, 34].

The study from Moster et al. reported higher neonatal mortality rates in hospitals with low birth volumes however, was lacking comparability between groups due to missing baseline-table and thus, quality was rated “unacceptable” [33]. In conclusion, methodically limitations hinder conclusive statements regarding the effect of birth volume on neonatal mortality.

Neonatal complications were reported in one study as a combined outcome (“perinatal adverse outcome”) including stillbirths, death ≤ 7 days, 5-min Apgar < 7 and a transfer to a neonatal intensive care unit in singleton births. Non-monotonous, significantly higher odds ratios of neonatal complications were reported for units with 750–999 and 1500–1749 births (Fig. 3) compared to at least 1750 births per anno [38].

### Effects of annual birth volume on maternal outcomes

Adjusted maternal mortality was reported as failing attempts to resuscitate women with severe complications during birth [28]. The volume-outcome relationships were reported to be non-monotonous in general with lower and higher relative risks of maternal mortality in lower (50) and higher annual birth volumes (≥ 2250–7500) [28].

**Fig. 4:**
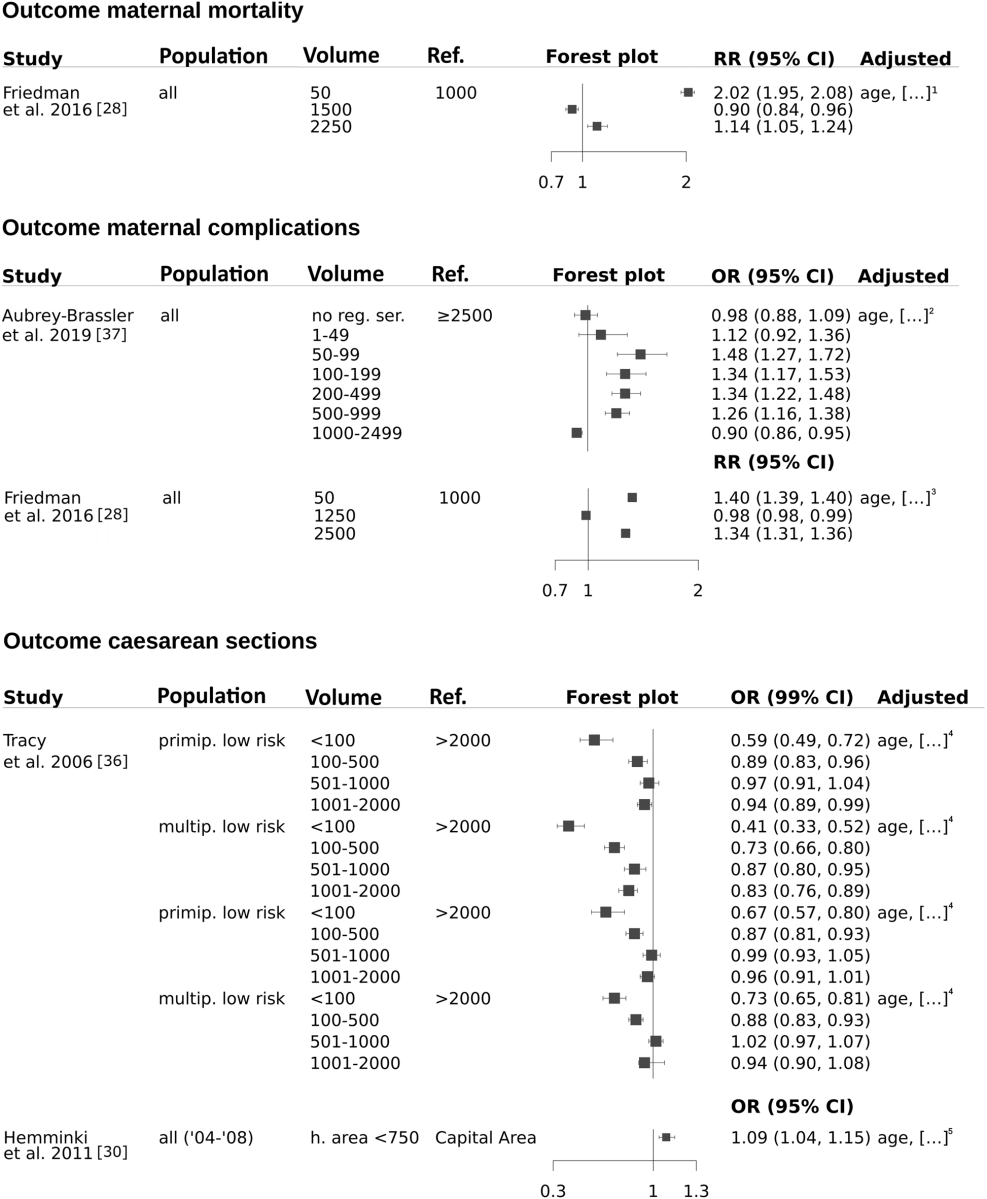
Maternal mortality, maternal complications and caesarean sections. Legend: […]1: race, hospital, year, comorbidity index, insurance status, household income, hospital teaching, hospital bed size, hospital region, hospital ownership, hospital location. […]2: GA, CS, Median income, Education rate, Aboriginal population, Unemployment rate, Minority, Statistical area classification, Travel Distance, Delivery hospital volume, Hospital level, HIV, Type 1/2 DM, Gestational/ other/ unspecified DM, Cystic fibrosis, Rheumatic heart disease, Hypertension, Ischemic heart disease, Pulmonary hypertension, SLE, Chronic renal disease, Twins/ multiple gestation, Previous CS. […]3: race, hospital, year, comorbidity index, insurance status, household income, hospital teaching, hospital bed size, hospital region, hospital ownership, hospital location. […]4: insurance status, maternal Aboriginal or Torres Strait Island status, maternal residential area. […]5: parity, smoking, socio-economic position

Adjusted maternal complications were reported in two studies as a combined outcome consisting of maternal mortality and different morbidy outcomes in all births [28, 37]. In a Canadian study the odds ratio were reported to be significantly higher in hospitals with ≤ 1000 births p.a [37]. However, a study from the US reported non-monotonous results with higher risk ratios in hospitals with high (2500) and low (50) annual birth volumes. Without providing results, the relative risks of maternal complications remained higher with a further increase in birth volume [28]. In conclusion, no conclusive statement regarding the impact of birth volume on maternal complication is possible due to contradicting study results as shown in Fig. 4.

An adjusted rate of delivery via caesarean section was reported in two studies [30, 36]. Hemminki et al. reported a significantly higher rate of caesarean sections in “small-hospital-areas” with less than 750 births per year compared to “capital areas” [30]. In contrast, Tracy et al. reported a significantly lower rate of caesarean sections in hospitals with ≤ 500 births [36]. Thus, contradicting study results do not allow conclusions regarding volume-effects on mode of delivery (Fig. 4).

In summary, most studies suggested a volume-outcome relationship on perinatal / early neonatal mortality and however reported either insignificant, non-monotonous or conflicting results regarding volume effects on the remaining outcomes.

## Discussion

This systematic review on the effects of hospital case volume on the safety and outcomes of infants classified as being on low risk births has tremendous public health impact, as births of children are so frequent and such an important life event. There is evidence already for high risk births and many other conditions such as preterm birth [1, 23], pediatric intensive care [71] or pediatric heart surgery [72] that hospitals with more experience and higher case numbers provide better healthcare indicated by better health outcomes of patients being treated there. We therefore speculated that higher birth volumes of hospitals were also related to better outcomes in births of low risk or all infants. These studies reported on mortality (stillbirths, perinatal, neonatal, maternal), morbidity (neonatal, maternal) and mode of delivery. Readmissions and developmental delays were not reported. Initially, a pooled estimate was intended. Heterogenities within the definitions and presentations of characteristics led to the decision not to perform a pooled estimate. Therefore, the results were synthesized qualitatively focusing on volume-outcome in general and especially in terms of lower annual birth volumes (≤ 1000). The heterogeneous results reported by two studies in different groups were not discussed by the study authors [30, 34] but might be caused by effect modifications.

While a possible effect of volume on early neonatal mortality was found to be consistent when statistical significance was reached, the influence of birth volume on other outcomes was less consistent. The reason for these inconsistencies has to be discussed. It could be assumed, that inconsistencies can be explained at a systemic level reflecting differences between national health care systems with variations in budgeting, access, geographical and historical conditions. One study included in this review showed differences of caesarean sections in dependence to hospital birth volume [36]. Several explanations could be discussed. It is possible that this could be an effect of perinatal regionalization treating high risk pregnancies in high birth volume hospitals leading into the need of surgical birth interventions. On the other hand, the appropriateness and need for the indication of e.g. epidural anesthesia was also discussed with reference to hospital ownership [15]. However, to further analyze the sensitive topic of appropriateness, qualitative research with primary data is needed. Due to the lack of detail information and data quality, routine data must be used with caution in order to avoid over- or misinterpretation [73].

With respect to a risk appropriate care, perinatal regionalization policies vary in terms of general organization, obligation and practice [2, 3]. At the provider level birth/delivery volumes may be only one covariate between several others such as time of birth, [38, 39, 70] personnel and material resources, [31, 32, 74] work environment [75] or qualifications [76] influencing the outcome of newborns indicated by studies included in this review.

Despite of lower early neonatal mortality in hospitals with high annual birth volume, closure of low volume institutions has to be considered very carefully, since reults have been discussed controversially. Some studies suggest a higher rate of unplanned out-of-hospital births [77] and an increased rate of neonatal mortality and stillbirths immediately after closures [58]. Furthermore, an increased rate of adverse birth outcomes [78] and higher stress/ anxiety levels of pregnant women were reported in large rural landscapes with long distances to access perinatal care [79]. Other studies report significantly lower rates of stillbirths and neonatal mortality in both rural and urban regions after closing maternity units [41].

The heterogeneous definitions identified in this and other systematic reviews [80] support the need for a standardized terminology of outcomes, populations and volume-thresholds. The definition of core-outcome sets (COS) would help to overcome that issue. The uniform terminology enables the design of comparable studies and forms the basis for the development of an international perinatal register. A homogeneously created perinatal register would allow individual patient data meta-analyses providing promising results as it has been shown for other indications [81, 82].

Overall most (12/13) of the included studies showed an “acceptable” quality as it is the highest rating for retrospective studies [26]. One study lacked an illustrated comparability of the study groups that led to “unacceptable” quality as it strongly limits transparency. None of the studies blinded the assesors nor was a report of non-blinding included. Nevertheless, we considered the studies as meaningful for interpretation because the assessed outcomes are difficult to manipulate and therefore the lack of blinding seems to be a minor weakness.

### Strengths and Limitations

This is the first systematic review explicitly assessing birth volume effects on neonatal outcome in low risk births. The review used transparent methods (independent screening, search strategy), was officially registered, is based on two major databases (combined with extensive hand search and expert panel for highlighting relevant literature) and followed common critical appraisal requirements of systematic reviews determined by AMSTAR 2 [83]. The high inter-rater-reliability ensures comprehensibility. The time and national restriction in the inclusion criteria could be interpreted as a limitation. However, it is well known that international comparisons must take into account the efficacy of health care systems [84, 85]. Thus, we used neonatal mortality rates as an indicator of this efficacy. With respect to the time restriction starting with publication in 2000, this review considered the decline of neonatal mortality and the development of perinatal care in since 1990 [86]. On the other hand, some of the studies have long past study periods (1967–2012) and intervals (1 to 29 years), indicating that the publication date did not work perfectly well as a delimiter to represent only current perinatal care. Almost every study showed an “acceptable” quality with retrospectively collected routine or register data.

## Conclusion

The aim of that review was originally to investigate volume-outcome associations in a comparatively low-risk birth cohort. With the exception of 7-day mortality, the review revealed heterogeneous results and major differences in the conception and definitions of the included studies.The qualitative synthesis of the studies indicated increased rates of early neonatal mortality (< 7d) in hospitals with birth volumes below 1000 or 500 births per anno when statistical significance was given. With respect to stillbirths, neonatal mortality, maternal mortality, caesarean section and neonatal and maternal complications the studies included reported inconclusive or insignificant results. Referring to the heterogeneously conducted study concepts in terms of assessed populations, volume-thresholds and outcomes, we recommend the development and use of internationally consented core-outcome sets to provide a homogenous definitional basis in future studies. A uniform terminology would enable a homogenously conceived internationally birth register for individual patient data meta analyses. Based on these data, strengths and weaknesses of different perinatal settings could be investigated using a common terminology of population, volume and outcome.

## Supplementary Information

**Additional file 1.**

**Additional file 2.**

**Additional file 3.**

**Additional file 4.**

**Additional file 5.**

## Data Availability

All data generated or analysed during this study are included in this published article [and its supplementary information files].

## Abbreviations

AUS: Australia
B-A: Before-After-Design
BMI: Body mass index
BW: Birth weight
CA: Canada
CI: Confidence interval
CS: Caesarean sections
d: Days
DM: Diabetes mellitus
FIN: Finland
GER: Germany
GA: Gestational age
h: Hospital
HIV: Humane immunodeficiency virus
NICU: Neonatal intensive care unit
NO: Norway
OR: Odds ratio
sv. morb: Severe morbidity
RR: Relative risk
p.a: Per anno
POR: Portugal
SCD: Sudden cardiac death
SIDS: Sudden infant death syndrome
SWE: Sweden
SLE: Systemic lupus erythematosus
UH: University hospital
wk: Week
UK: United Kingdom
US: United States
