# Supplementary material for: Are birth outcomes in low risk birth cohorts related to hospital birth volumes? A systematic review
Source: BMC Pregnancy Childbirth. 2021 Jul 27;21:531. doi: 10.1186/s12884-021-03988-y (PMC8314545; doi:10.1186/s12884-021-03988-y)
Supplement: Supplementary file 1 — Additional file 1. [file 12884_2021_3988_MOESM1_ESM.docx]

# Additional file 1 - Search strategy per database

|  | **Medline: 4475 Records** | **Embase: 6448 Records** |
| --- | --- | --- |
| 1 | Meta-Analysis as Topic/ or meta analy$.tw. or metaanaly$.tw. or Meta-Analysis/ or (systematic adj (review$1 or overview$1)).tw. or exp Review Literature as Topic/ | exp Meta Analysis/ or ((meta adj analy$) or metaanalys$).tw. or (systematic adj (review$1 or overview$1)).tw. |
| 2 | (cochrane or embase or (psychlit or psyclit) or (psychinfo or psycinfo) or (cinahl or cinhal) or science citation index or bids or cancerlit).ab. | cancerlit.ab. or cochrane.ab. or embase.ab. or medline.ab. or (psychlit or psyclit).ab. or (psychinfo or psycinfo).ab. or (cinahl or cinhal).ab. or science citation index.ab. or bids.ab |
| 3 | (reference list$ or bibliograph$ or hand-search$ or relevant journals or manual search$).ab. | reference lists.ab.or bibliograph$.ab. or hand-search$.ab. or manual search$.ab. or relevant journals.ab. or relevant articles.ab. or relevant studies.ab. |
| 4 | (selection criteria or data extraction).ab. and Review/ | (data extraction.ab. or selection criteria.ab. or inclusion criteria.ab.) and review.pt. |
| 5 | (comment/ or letter/ or Editorial/ or animal/) not (animal/ and human/) | letter.pt.or editorial.pt. or animal/ not (animal/ and human/) |
| 6 | 1 or 2 or 3 or 4 | 1 or 2 or 3 or 4 |
| 7 | 6 not 5 | 6 not 5 |
| 8 | infant/ or infant, newborn/ or parturition/ or natural childbirth/ or term birth/ or Birth Weight/ or (term birth or normal birth or normal birth weight or normal birthweight or low risk birth).ti. or (term birth or normal birth or normal birth weight or normal birthweight or low risk birth).ab. | infant/ or newborn/ or birth weight/ or high birth weight/ or term birth/ or childbirth/ or natural childbirth/ or (term birth or normal birth or normal birth weight or normal birthweight or low risk birth).ti. or (term birth or normal birth or normal birth weight or normal birthweight or low risk birth).ab. |
| 9 | ((perinatal* or matern* or obstetric* or newborn* or neonatal*) AND (care or hospital$ or unit or facility)).ti. or ((perinatal* or matern* or obstetric* or newborn* or neonatal*) and (care or hospital$ or unit or facility)).ab. or nicu.ti. or nicu.ab. or (neonatal and icu).ti. or (neonatal and icu).ab. or perinatal care/ or neonatal intensive care unit/ | ((perinatal* or matern* or obstetric* or newborn* or neonatal*) AND (care or hospital$ or unit or facility)).ti. or ((perinatal* or matern* or obstetric* or newborn* or neonatal*) and (care or hospital$ or unit or facility)).ab. or nicu.ti. or nicu.ab. or (neonatal and icu).ti. or (neonatal and icu).ab. or perinatal care/ or postnatal care/ or prenatal care/ or neonatal intensive care unit/ or pediatrics/ or neonatology/ or obstetrics/ or perinatology/ |
| 10 | (region$ or central$ or urban or rural or Volume$ or size or level or type or caseload or case load).ti. or (region$ or central$ or urban or rural or Volume$ or size or level or type or caseload or case load).ab. or health facility size/ or hospitals, high-volume/ or hospitals, low-vo­lume/ or hospitals, public/ or hospitals, rural/ or hospitals, maternity/ or hospitals, pediatric/ | (region$ or central$ or urban or rural Volume$ or size or level or type or caseload or case load).ti. or (region$ or central$ or urban or rural Volume$ or size or level or type or caseload or case load).ab. or regionalization/ or high volume hospital/ or low volume hospital/ |
| 11 | hospital mortality/ or infant mortality/ or mortality/ or newborn mortality/ or prenatal mortality/ or perinatal mortality/ or fetus mortality/ or maternal mortality/ or ((neonatal* or perinatal* or matern*) and (death or mortality)).ti. or ((neonatal* or perinatal* or matern*) and (death or mortality)).ab. | hospital mortality/ or mortality/ or perinatal mortality/ or newborn mortality/ or fetus mortality/ or premature mortality/ or prenatal mortality/ or maternal mortality/ or ((neonatal* or perinatal* or matern*) and (death or mortality)).ti. or ((neonatal* or perinatal* or matern*) and (death or mortality)).ab. |
| 12 | 8 AND 9 AND 10 not 7 or (case reports/ or clinical conference/ or consensus development conference/ or consensus development conference, nih/ or validation studies/) | (8 and 9 and 10 and 11) not (7 or case report/ or consensus development/ or practice guideline/ or in vitro study/ or preclinical study/ or in vivo study/ or validation study/ or case study/ |
|  | (Filter 2000, english/ german) | (Filter 2000, english/ german) |
