# Supplementary material for: Are birth outcomes in low risk birth cohorts related to hospital birth volumes? A systematic review
Source: BMC Pregnancy Childbirth. 2021 Jul 27;21:531. doi: 10.1186/s12884-021-03988-y (PMC8314545; doi:10.1186/s12884-021-03988-y)
Supplement: Supplementary file 2 — Additional file 2. [file 12884_2021_3988_MOESM2_ESM.docx]

# Additional file 2 - Methods and definitions using CASP checklist for cohort studies

Item 1.1 adresses the presence of a suitable and clearly focused reseach question as it is necessary to assess the relevance and value of the reported results.

Due to a possible selection bias, the study groups should have as similar characteristics as possible to guarantee a comparability (1.2) and the study needs to report the proportion of invited and participating participants for the entire study and study arm (1.3).

Item 1.4 queries the probability or the performing of sensitivity analyses that a participant already has the result at the beginning of the study to identify performance bias.

To address a possible attrition bias item 1.5 and 1.6 query the drop-out and lost to follow-up-rates to detect a possible violation of a representative study sample.

The items 1.7 to 1.12 deal with a transparent and consistent execution of the study with regard to a possible detection bias. This includes clearly defined and consequently measured outcomes (1.7), blinding (1.8) and the reported influence of non-blinding (1.9). To detect the influence of non-blinding a study process analysis (e.g. number of observations, observators) is recommended. In terms of measurement of exposure level, the measurement method should be comprehensibly reported (1.10.) and the measurement itself should be carried out multpiple times (1.12). With regard to the study results, a valid presentation must include a traceable/referenced measurement (1.11), identify and statistically consider possible confounders (1.13) and report confidence intervals (1.14). These items are crucial for the validity, bias and reliability of the outcomes presented.

It should be taken into account that the items 1.3, 1.5, 1.6 and 1.12 are only queried for prospective studies. Consequently, only prospective studies can receive a "high quality" rating, since all criteria must be fulfilled for this rating.[[26](#_ENREF_28)] An “acceptable” quality was given if at least item 1.1 and 1.2 were fulfilled with regards to a minimum standard of replicability and transparency. Otherwise the study was rated with “unacceptable” quality.
