# Supplementary material for: Are birth outcomes in low risk birth cohorts related to hospital birth volumes? A systematic review
Source: BMC Pregnancy Childbirth. 2021 Jul 27;21:531. doi: 10.1186/s12884-021-03988-y (PMC8314545; doi:10.1186/s12884-021-03988-y)
Supplement: Supplementary file 3 — Additional file 3. [file 12884_2021_3988_MOESM3_ESM.docx]

# Additional file 3 - Excluded studies with reasons

| Reference | Reason f. Exclusion |
| --- | --- |
| Adams N, Tudehope D, Gibbons KS, Flenady V: Perinatal mortality disparities between public care and private obstetrician-led care: a propensity score analysis. BJOG : an international journal of obstetrics and gynaecology 2018, 125(2):149-158. | No volume-outcome |
| Allen VM, Jilwah N, Joseph KS, Dodds L, O'Connell CM, Luther ER, Fahey TJ, Attenborough R, Allen AC: The influence of hospital closures in Nova Scotia on perinatal outcomes. Journal of obstetrics and gynaecology Canada : JOGC = Journal d'obstetrique et gynecologie du Canada : JOGC 2004, 26(12):1077-1085. | No volume-outcome |
| Badheka A, Rampa S, Wang T, Nalliah R, Caplin J, Allareddy V: Neonatal infections in Hospitals: Nationwide prevalence and outcomes. Critical Care Medicine 2019, 47(1 Supplement 1). | No mortality measured |
| Clapp MA, James KE, Bates SV, Kaimal AJ: Patient and Hospital Factors Associated With Unexpected Newborn Complications Among Term Neonates in US Hospitals. JAMA network open 2020, 3(2):e1919498. | No mortality measured |
| Engjom H, Morken NH, Hoydal E, Norheim OF, Klungsoyr K: Obstetric health system structure and perinatal outcomes in Norway. International Journal of Gynecology and Obstetrics 2015, 131(SUPPL. 5):E487-E488. | Conference Abstract |
| Engjom H, Moster D, Morken NH, Hoydahl E, Norheim OF, Klungsoyr K: Perinatal mortality and health system structure in Norway-a population-based registry study. BJOG: An International Journal of Obstetrics and Gynaecology 2016, 123(Supplement 2):11. | Conference Abstract |
| Filipovic-Grcic B, Kniewald H, Rodin U, Grizelj R, Stipanovic-Kastelic J, Ninkovic D, Gveric-Ahmetasevic S, Stanojevic M, Furlan IA, Peter B et al: Patterns of newborns' deaths to discharge from hospital in Croatia in the year 2011. Gynaecologia et Perinatologia 2012, 21(SUUPL.1):150-156. | Croatian language |
| Grytten J, Monkerud L, Skau I, Sorensen R: Regionalization and local hospital closure in Norwegian maternity care--the effect on neonatal and infant mortality. Health services research 2014, 49(4):1184-1204. | No volume-outcome |
| Heller G, Schnell R, Richardson DK, Misselwitz B, Schmidt S: [Assessing the impact of delivery unit size on neonatal survival: estimation of potentially avoidable deaths in Hessen, Germany, 1990-2000]. Hat die Grosse der Geburtsklinik Einfluss auf das neonatale Uberleben? Schatzung von "vermeidbaren" Todesfallen in Hessen 1990-2000 2003, 128(13):657-662. | Descriptive study |
| Homer CSE, Thornton C, Scarf VL, Ellwood DA, Oats JJN, Foureur MJ, Sibbritt D, McLachlan HL, Forster DA, Dahlen HG: Birthplace in New South Wales, Australia: an analysis of perinatal outcomes using routinely collected data. BMC pregnancy and childbirth 2014, 14:206. | No Comparison of hospitals |
| Hughes S, Zweifler JA, Garza A, Stanich MA: Trends in rural and urban deliveries and vaginal births: California 1998-2002. The Journal of rural health : official journal of the American Rural Health Association and the National Rural Health Care Association 2008, 24(4):416-422. | No Comparison of hospitals |
| Hurtado Suazo JA, Demestre Guasch X, Garcia Reymundo M, Ginovart Galiana G, Gimenez A, Calvo Aguilar MJ, Trincado Aguinagalde MJ, Fernandez Colomer B: Comparison of perinatal data between a cohort of Spanish late preterm babies and another of term newborns. Journal of Perinatal Medicine 2015, 43(SUPPL. 1). | Conference Abstract |
| Iglesias S, Bott N, Ellehoj E, Yee J, Jennissen B, Bunnah T, Schopflocher D: Outcomes of maternity care services in Alberta, 1999 and 2000: a population-based analysis. Journal of obstetrics and gynaecology Canada : JOGC = Journal d'obstetrique et gynecologie du Canada : JOGC 2005, 27(9):855-863. | No Comparison of hospitals |
| Karalis E, Gissler M, Tapper AM, Ulander VM: Influence of time of delivery on risk of adverse neonatal outcome in different size of delivery units: a retrospective cohort study in Finland. Journal of Maternal-Fetal and Neonatal Medicine 2019, 32(10):1696-1702. | Full-text not available |
| Koch R, Gmyrek D, Vogtmann C: [Risk adjusted assessment of quality of perinatal centers - results of perinatal/neonatal quality surveillance in Saxonia]. Risikoadjustierte Qualitatsbeurteilung in Perinatalzentren ausgehend von der Perinatal- und Neonatalerhebung in Sachsen 2005, 209(6):210-218. | No Comparison of hospitals |
| Kozhimannil KB, Interrante JD, Henning-Smith C, Admon LK: Rural-Urban Differences In Severe Maternal Morbidity And Mortality In The US, 2007-15. Health affairs (Project Hope) 2019, 38(12):2077-2085. | No volume-outcome |
| Krzyzak M, Maslach D, Piotrowska K, Charkiweicz AE, Szpak A, Karczewski J: Perinatal mortality in urban and rural areas in Poland in 2002-2012. Przeglad epidemiologiczny 2014, 68(4):675-679. | No Comparison of hospitals |
| Lesniczak B, Krasomski G, Rudnicka B, Piekarska E, Oszukowski P, Wozniak P: The perinatal mortality of fetuses and neonates in Poland in the years 1960-2010. Ginekologia i Poloznictwo 2015, 36(2):40-45. | No Comparison of hospitals |
| Lorch SA, Srinivas SK, Ahlberg C, Small DS: The impact of obstetric unit closures on maternal and infant pregnancy outcomes. Health services research 2013, 48(2 Pt 1):455-475. | No volume-outcome |
| Neto MT: Perinatal care in Portugal: effects of 15 years of a regionalized system. Acta paediatrica (Oslo, Norway : 1992) 2006, 95(11):1349-1352. | Descriptive study |
| Parazzini F, Cipriani S, Bulfoni G, Bulfoni C, Bellu R, Zanini R, Mosca F: Mode of delivery and level of neonatal care in Lombardy: a descriptive analysis according to volume of care. Italian journal of pediatrics 2015, 41:24. | No mortality measured |
| Payne JC, Campbell MK, DaSilva O, Koval J: Perinatal mortality in term and preterm twin and singleton births. Twin research : the official journal of the International Society for Twin Studies 2002, 5(4):260-264. | No volume-outcome |
| Poeran J, Borsboom GJJM, de Graaf JP, Birnie E, Steegers EAP, Mackenbach JP, Bonsel GJ: Does centralisation of acute obstetric care reduce intrapartum and first-week mortality? An empirical study of over 1 million births in the Netherlands. Health policy (Amsterdam, Netherlands) 2014, 117(1):28-38. | Modelling |
| Ravelli ACJ, Tromp M, van Huis M, Steegers EAP, Tamminga P, Eskes M, Bonsel GJ: Decreasing perinatal mortality in The Netherlands, 2000-2006: a record linkage study. Journal of epidemiology and community health 2009, 63(9):761-765. | No Comparison of hospitals |
| Reid LD, Creanga AA: Severe maternal morbidity and related hospital quality measures in Maryland. Journal of Perinatology 2018, 38(8):997-1008. | No mortality measured |
| Shuvalova MP, Yarotskaya EL, Pismenskaya TV, Dolgushina NV, Baibarina EN, Sukhikh GT: Maternity Care in Russia: Issues, Achievements, and Potential. Journal of obstetrics and gynaecology Canada : JOGC = Journal d'obstetrique et gynecologie du Canada : JOGC 2015, 37(10):865-871. | Descriptive study |
| Treurniet HF, Looman CW, van der Maas PJ, Mackenbach JP: Regional trend variations in infant mortality due to perinatal conditions in the Netherlands. European journal of obstetrics, gynecology, and reproductive biology 2000, 91(1):43-49. | No Comparison of hospitals |
| Harvey SM, Oakley LP, Yoon J, Luck J: Coordinated Care Organizations: Neonatal and Infant Outcomes in Oregon. Medical Care Research and Review 2019, 76(5):627-642. | Perinatal Regionalization |
| Merlo J, Gerdtham U-G, Eckerlund I, Hakansson S, Otterblad-Olausson P, Pakkanen M, Lindqvist P-G: Hospital level of care and neonatal mortality in low- and high-risk deliveries: reassessing the question in Sweden by multilevel analysis. Medical care 2005, 43(11):1092-1100. | Perinatal Regionalization |
| Serenius F, Winbo I, Dahiquist G, Kallen B: Cause-specific stillbirth and neonatal death in Sweden: a catchment area-based analysis. Acta paediatrica (Oslo, Norway : 1992) 2001, 90(9):1054-1061. | Perinatal Regionalization |
