# Supplementary material for: Are birth outcomes in low risk birth cohorts related to hospital birth volumes? A systematic review
Source: BMC Pregnancy Childbirth. 2021 Jul 27;21:531. doi: 10.1186/s12884-021-03988-y (PMC8314545; doi:10.1186/s12884-021-03988-y)
Supplement: Supplementary file 4 — Additional file 4. [file 12884_2021_3988_MOESM4_ESM.docx]

# Additional file 4 - Information on funding, conflict of interest and datasources within the included studies

| Ref. | Funding | CoI | Data­sources |
| --- | --- | --- | --- |
| Finnstrom et al. 2006[[2](#_ENREF_29)*7*] | N/A | N/A | Swedish Medical Birth Registry and the Hospital Discharge Registry |
| Friedman et al. 2016[28] | public | n | Nationwide Inpatient Sample (NIS) |
| Heller et al. 2002[29] | N/A | N/A | perinatal birth register |
| Hemminki et al. 2011[30] | no grant received | n | medical birth register data |
| Joyce et al. 2004[[3](#_ENREF_33)1] | N/A | N/A | Office for Natio­nal Statistics (ONS) birth and death registrations, the 1991 Census, Royal College of Ob­stetricians and Gynaecologists (RCOG) hosp­ital reco­gnition returns, De­partment of Health (DOH) data on hospital staffing levels, survey of risk management practices in Thames maternity units |
| Karalis et al. 2016[[3](#_ENREF_34)2] | no grant received | n | National Medical Birth Register |
| Moster et al. 2001[[3](#_ENREF_35)3] | public | N/A | The Norwegian Medical Birth Registry |
| Pyykonen et al. 2014[[3](#_ENREF_36)4] | no grant received | n | Medical Birth Register data |
| Snowden et al. 2012[[3](#_ENREF_37)5] | N/A | n | linked birth/infant death certiﬁcates with hospital discharge diagnoses for births |
| Tracy et al. 2006[[3](#_ENREF_38)6] | public | n | National Perinatal Data Collection (NPDC) |
| de Graaf et al. 2010[[3](#_ENREF_40)8] | no grant received | none | Netherlands Perinatal Registry, a linked professional database of all pregnancies, of 20 weeks and above, in the Netherlands, collected from (referring) midwives, obstetricians and paediatricians. |
| Restrepo et al. 2018 [39] | public | none | electronic birth certificate records matched with 92 death certificate records from the Texas Department of State Health Services |
| Aubrey-brassler et al. 2019[37] | public | none | maternal and neonatal delivery hospitalization records were accessed from the Canadian Institute for Health Information (CIHI) |

Notes: BMI: body mass index d: days
