# Supplementary material for: Are birth outcomes in low risk birth cohorts related to hospital birth volumes? A systematic review
Source: BMC Pregnancy Childbirth. 2021 Jul 27;21:531. doi: 10.1186/s12884-021-03988-y (PMC8314545; doi:10.1186/s12884-021-03988-y)
Supplement: Supplementary file 5 — Additional file 5. [file 12884_2021_3988_MOESM5_ESM.docx]

# Additional file 5 Methods and definitions of the outcomes assessed

| **Reference** | **Outcome definition** | **Population** | **Grouped Hospital Volume** | | **Estimator** | | **Adjusted** | | |
| --- | --- | --- | --- | --- | --- | --- | --- | --- | --- |
| Stillbirths |  |  |  | |  | |  | | |
| Karalis et al. 2016[[3](#_ENREF_34)2] | - | low risk | births: ≤999, 1000-1999, ≥2000, UH (ref.) | | OR (95% CI) | | age, parity | | |
| Pyykonen et al. 2014[[3](#_ENREF_36)4] | >22wk GA | term + all (UH excl.) | women: <1000, 1000-2999 (ref.), <3000 | | OR (95% CI) | | - | | |
| Joyce et al. 2004[[3](#_ENREF_33)1] | >24wk GA | all | births: Mean/ year (no reference volume) | | Slope | | BW | | |
| Perinatal/ Early neonatal mortality | | |  | | |  | |  |  |
| Heller et al. 2002[29] | ≤7d | BW >2500g, BW >2500g + term | births: ≤500, 501-1000, 1001-1500, >1500 (ref.) | | OR (95% CI) | | GA, BW, age, parity, born outside clinic, birth planned and documented clinic, mode of delivery, born before arrival at clinic, time of birth, congenital anomaly/ malformation | | |
| Pyykonen et al. 2014[[3](#_ENREF_36)4] | Stillbirth/death≤7d, death≤7d | All + term, UH excl. | women: <1000, 1000-2999 (ref.), <3000 | | OR (95% CI) | | - | | |
| Hemminki et al. 2011[[3](#_ENREF_32)0] | ≤7d | all, BW>2499g | births: <750, 750-1499, ≥1500, UH (ref.) | | OR (95% CI) | | BW, age, parity, socio-economic position | | |
| Karalis et al. 2016[[3](#_ENREF_34)2] | - | low risk | births: ≤999, 1000-1999, ≥2000, UH (ref.) | | OR (95% CI) | | age, parity | | |
| de Graaf et al. 2010[38] | ≤7d | Singleton | women: <750, 750-999, 1000-1249, 1250-1499, 1500-1749, ≥1750 (ref.) | | OR (95% CI) | | GA, age, parity, mode of delivery, ethnicity, calendar year trend | | |
| Aubrey-Brassler et al. 2019[[3](#_ENREF_39)7] | in-hospital, stillbirth (GA≤20wk), SIDS, SCD | All | women: No services usually; 1-49; 50-99; 100-199; 200-499; 500-999; 1000-2499, >2500 (ref.) | | OR (95% CI) | | BW, gender, Eclampsia, Premature rupture of membranes, Oligohydramnios, Abruptio placentae, Prolapsed umbilical cord, Noxious influences transmitted via placenta/ breast milk, Congenital anomalies, Hydrops fetalis, Other maternal conditions, | | |
| Neonatal mortality | |  | |  | |  | |  | |
| Finnstrom et al. 2006[[2](#_ENREF_29)7] | ≤27d | Singleton | births: <500, 500-999, 1000-2499 (ref.), ≥2500 | | OR (95% CI) | | age, parity, GA, year of birth, smoking, parental cohabitation, maternal BMI | | |
| Moster et al. 2001[[3](#_ENREF_35)3] | ≤28d | BW>2.499g | births: ≤100, 101-500, 501-1000, 1001-2000, 2001-3000, >3000 (ref.) | | OR (95% CI) | | birth year | | |
| Pyykonen et al. 2014[[3](#_ENREF_36)4] | ≤28d | term, all, UH excl. | women: <1000, 1000-2999 (ref.), <3000 | | OR (95% CI) | | - | | |
| Tracy et al. 2006[[3](#_ENREF_38)6] | ≤28d | Primip. low risk,  multip. low risk | births: <100, 100-500, 501-1000, 1001-2000 >2001 (ref.) | | OR (99% CI) | | age, insurance status, maternal Aboriginal or Torres Strait Island status, maternal residential area | | |
| Joyce et al. 2004[[3](#_ENREF_33)1] | ≤28d | All | births: Mean/ year (no reference volume) | | Slope | | BW | | |
| Snowden et al. 2012[[3](#_ENREF_37)5] | - | Urban: all, low risk rural: all, low risk | women: Urban: ≤50-1199 (ref.), 1200-2399, 2400-3599; ≥3600  Rural: 50-599 (ref.) 600-1699; ≥1700 | | Rate | | - | | |
| Restrepo et al. 2018[39] | ≤28d | All | births: linear trend (no reference volume) | | Pearson test | | - | | |
| neonatal complications | |  | |  | |  | |  | |
| de Graaf et al. 2010[38] | Stillbirth/ death≤7d, 5-min. Apgar<7, NICU transfer | Singleton | women: <750, 750-999, 1000-1249, 1250-1499, 1500-1749, ≥1750 (ref.) | | OR (95% CI) | | GA, age, parity, mode of delivery, ethnicity, calendar year trend | | |
| maternal mortality | |  | |  | |  | |  | |
| Friedman et al. 2016[28] | failed rescue | all | women: 50, 1000 (ref.), 1500, 2250 | | RR (95 % CI) | | age, race, hospital, year, comorbidity index, insurance status, household income, hospital teaching, hospital bed size, hospital region, hospital ownership, hospital location | | |
| maternal complications | |  | |  | |  | |  | |
| Aubrey-Brassler et al. 2019[[3](#_ENREF_39)7] | Eclampsia, Previa with hemorrhage abruption, Intrapartum + postpartum hemorrhage + transfusion or hysterectomy, Rupture of uterus before or during labor, Obstetric shock, Sepsis, Other complications of obstetric procedures, Obstetric embolism, Cardiovascular disease, Acute renal failure, Death, obstetric or unspecified, Neurologic disease, Hematologic disease, Respiratory disease, Diabetic ketoaci-dosis, Peritonitis or parametritis, Toxic liver disease or hepatic failure, Canadian Classification of Health Interventions, Assisted ventilation or resuscitation, Dialysis, Hysterectomy, Evacuation of incisional hemato-ma, Repair of bladder, urethra or intestine, Embolization or ligation of pelvic vessels or suturing of uterus, Blood transfusion | all | women: No services usually; 1-49; 50-99; 100-199; 200-499; 500-999; 1000-2499, >2500 (ref.) | | OR (95% CI) | | age, GA, CS, Median income, Education rate, Aboriginal population, Unemployment rate, Minority, Statistical area classification, Travel Distance, Delivery hospital volume, Hospital level, HIV, Type 1/2 DM, Gestational/ other/ unspecified DM, Cystic fibrosis, Rheumatic heart disease, Hypertension, Ischemic heart disease, Pulmonary hypertension, SLE, Chronic renal disease, Twins/ multiple gestation, Previous CS | | |
| Friedman et al. 2016[28] | heart/ renal/ respiratory failure, acute myocardial infarction, liver disease, disseminated intravascular coagulation, coma, delirium, puerperal cerebrovascular disorders, pulmonary edema or embolism, sepsis, shock, status asthmaticus, status epilepticus. | all | women 50, 1250 2500 | | RR (95 % CI) | | age, race, hospital, year, comorbidity index, insurance status, household income, hospital teaching, hospital bed size, hospital region, hospital ownership, hospital location | | |
| caesarean sections | |  | |  | |  | |  | |
| Tracy et al. 2006[[36](#_ENREF_38)] | labour, all | Primip. low risk, multip. low risk | births: <100, 100-500, 501-1000, 1001-2000 >2001 (ref.) | | OR (99% CI) | | age, insurance status, maternal Aboriginal or Torres Strait Island status, maternal residential area | | |
| Hemminki et al. 2011[30] | - | all | h. area <750 births p.a., capital area (ref.) | | OR (95% CI) | | age, parity, smoking, socio-economic position | | |
